# Characterization of Endolysin LysECP26 Derived from rV5-like Phage vB_EcoM-ECP26 for Inactivation of *Escherichia coli* O157:H7

**DOI:** 10.4014/jmb.2005.05030

**Published:** 2020-07-17

**Authors:** Do-Won Park, Jong-Hyun Park

**Affiliations:** Department of Food Science and Biotechnology, Gachon University, Seongnam 13120, Republic of Korea

**Keywords:** *E. coli* O157:H7, rV5-like phage, endolysin, outer membrane permeabilizers (OMPs)

## Abstract

With an increase in the consumption of non-heated fresh food, foodborne shiga toxin-producing *Escherichia coli* (STEC) has emerged as one of the most problematic pathogens worldwide. Endolysin, a bacteriophage-derived lysis protein, is able to lyse the target bacteria without any special resistance, and thus has been garnering interest as a powerful antimicrobial agent. In this study, rV5-like phage endolysin targeting *E. coli* O157:H7, named as LysECP26, was identified and purified. This endolysin had a lysozyme-like catalytic domain, but differed markedly from the sequence of lambda phage endolysin. LysECP26 exhibited strong activity with a broad lytic spectrum against various gram-negative strains (29/29) and was relatively stable at a broad temperature range (4°C– 55°C). The optimum temperature and pH ranges of LysECP26 were identified at 37°C–42°C and pH 7– 8, respectively. NaCl supplementation did not affect the lytic activity. Although LysECP26 was limited in that it could not pass the outer membrane, *E. coli* O157: H7 could be effectively controlled by adding ethylenediaminetetraacetic acid (EDTA) and citric acid (1.44 and 1.14 log CFU/ml) within 30 min. Therefore, LysECP26 may serv as an effective biocontrol agent for gram-negative pathogens, including *E. coli* O157:H7.

## Introduction

*Escherichia coli*, a gram negative-bacterium, is commonly found in the gut microbiota and plays an important role in digestive processes [1]. However, shiga toxin-producing *E. coli* (STEC) strains (mainly serotype O157:H7) can cause hemolytic uremic syndrome and acute renal injury [2]. Controlling STEC in ready-to-eat food has emerged as an important issue owing to both the importance and difficulty of this undertaking [3].

Bacteriophages (phages) are host specific bacteria-infecting viruses that are non-toxic in humans and effective tools for combatting antibiotic-resistant pathogens [4, 5]. Because of these advantages, the process of isolating and identifying phages to control STEC has been steadily progressing, and the application of phages to various foods has shown significant results [6-9]. In particular, the recently classified and identified “rV5-like phage,” a member of the myovirus genus that shows strong antibacterial activity against *E. coli* O157: H7 and has a broad host spectrum, has received attention as a potential agent to control STEC [10-12]. Nevertheless, previous studies have mostly focused on λ, T-even, and P1-like phages, so the “rV5-like phage” genus requires additional study [13].

Phage-derived peptidoglycan hydrolase “endolysin” has also been suggested as an effective antimicrobial agent [14]. Phages are clearly powerful antimicrobial agents; however, some pathogens can resist phages through restriction enzymes or the CRISPR-Cas system [15]. In contrast, endolysin is considered to be more efficient than phages as it exerts its bactericidal effect by simply degrading peptidoglycan linkages [16]. Therefore, direct treatment with purified endolysin has been known to be highly effective. However, this strategy is only effective on gram-positive pathogens; its effects on gram-negative pathogens are limited because of the protective outer membrane (OM) [14]. To overcome this problem, strategies such as combining the hydrophobic peptide with endolysin or treatment with OM-permeabilizers (OMPs) such as ethylenediaminetetraacetic acid (EDTA) and organic acid have been attempted [17].

There have been several annotation reports of *E. coli* phages belonging to the rV5-like phage genus; however, to our knowledge, studies on the expression and characterization of endolysin have not been reported. Previously, the *E. coli* O157:H7-infecting phage vB_EcoM-ECP26, which belongs to the rV5-like phage group, was isolated and characterized, and its genome was completely sequenced [18]. In this study, using a bioinformatics approach, endolysin gene derived from vB_EcoM-ECP26 was analyzed. This endolysin, named LysECP26, was isolated and characterized by its biochemical properties. LysECP26 showed lytic activity against various gram-negative bacteria, as well as pathogenic *E. coli* strains. In addition, a combination of LysECP26 and OMPs could control STEC to a significant level, suggesting that it could contribute to controlling STEC in the food industry.

## Materials and Methods

### Bioinformatic Analysis

LysECP26 has been previously identified as a phage endolysin. BLASTP analysis was performed to determine homologous proteins within the LysECP26 sequence [19]. A domain search was performed using the NCBI’s Conserved Domain Database (CDD) [20]. Amino acid sequences of LysECP26 and several known endolysins were aligned using Clustal Omega [21]. The GenBank accession numbers of phage endolysins used in this study are as follows: LysECP26 (QDB73524.1), lambda (AAA96598.1), T4 (AAD42568.1), rV5 (YP_009177530.1), FV3 (YP_007006253.1), 203 (ATW61430.1).

### Bacterial Strains and Growth Conditions

The bacterial strains used in this study are described in Table S1. All strains were cultured in Luria Bertani (LB) broth (Difco, USA) at 37°C and 150 rpm. When needed, 50 μg/ml of ampicillin or 12.5 μg/ml of chloramphenicol was added to the growth medium.

### Plasmid Construction and Purification of Endolysin LysECP26

Purified genomic DNA of phage vB_EcoM-ECP26 was used as a template for PCR, and the LysECP26 gene was amplified using the following primers: LysECP26_F (5'-GGAATTCCAT ATGAAACTTGATAAAAATGTT-3') and LysECP26_R (5'-GCCCTCGAGATCTAGGAC AA-3') (restriction sites are underlined). The amplified PCR product was digested with NdeI and XhoI enzymes and ligated into the pET23a (Novagen, USA), which contained a C-terminal hexahistidine (6× His)-tag sequence. The constructed plasmid was transformed into pRARE containing *E. coli* C41 (DE3) for LysECP26 expression [22]. The transformant was grown until it reached an O.D_600_ of 0.5–0.6 in LB broth supplemented with ampicillin (final conc.: 50 μg/ml) and chloramphenicol (final conc.: 12.5 μg/ml). It was then induced by the addition of 1 mM isopropyl β-D-1-thiogalactopyranoside, followed by incubation at 37°C for 4 h with shaking [23]. The induced *E. coli* were harvested by centrifugation at 7,800 g for 10 min, resuspended in lysis buffer (20 mM Tris-HCl, 300 mM NaCl, pH 8.0), and disrupted by sonication on ice for 45 min (5/2 s pulse on/off, Amp 40%) using a model VCX130 device (Vibra-Cell, USA). The lysate debris was removed by centrifugation at 14,000 rpm for 10 min, and soluble proteins were obtained by filtration (0.22 μm pore size; Milipore, USA). Recombinant proteins were purified using a Ni-NTA Superflow resin (Qiagen, Germany), according to the manufacturer’s instructions. Sodium dodecyl sulfate-polyacrylamide gel electrophoresis (SDS-PAGE) was conducted to identify the purified endolysin. Purified protein solution was concentrated and dialyzed against the storage buffer (20 mM Tris-HCl, 300 mM NaCl, 30% glycerol, pH 8.0) using an Amicon Ultra-4 device (Millipore). Protein concentration was determined using the Bradford reagent (Bio-Rad Laboratories, USA) according to the manufacturer's instructions. The dialyzed protein was stored at -80°C.

### Antimicrobial Activity of LysECP26

To confirm the bactericidal activity of LysECP26, exponentially grown *E. coli* DH5α cells were harvested and suspended in EDTA-containing buffer (20 mM Tris-HCl, 0.1 M EDTA, pH 8.0) for 5 min. After centrifugation to remove the EDTA, the cell pellets were resuspended two times with reaction buffer (20 mM Tris-HCl, pH 8.0). LysECP26 was serially diluted and inoculated into the washed *E. coli* (final conc.: 1–1,000 ng/ml) culture. The same volume of reaction buffer was used as negative control. Reductions in O.D_600_ values were measured after 10 min [24].

### Antimicrobial Spectrum of LysECP26

The antimicrobial spectrum of LysECP26 was tested against six gram-positive strains and 29 gram-negative strains. All exponentially grown bacteria were pretreated in the same manner as in the antimicrobial activity assay. LysECP26 (final conc. 1,000 ng/ml) or the reaction buffer was added to the washed bacteria culture for 30 min at 37°C. The experiments were replicated three times. Comparison of the O.D_600_ values for the reaction-buffer-only treatment group and the LysECP26 treatment group revealed that lysis occurred when statistically significant results appeared (*p* < 0.01).

### Characterization of LysECP26

To evaluate the temperature stability of LysECP26, samples of LysECP26 (final conc. 1,000 ng/ml) were incubated at different temperatures (4°C, 25°C, 37°C, 42°C, 55°C, and 72°C) for 30 min [25]. Non-incubated endolysin served as a positive control. After each treatment, endolysin was incubated with EDTA-treated *E. coli* DH5α cells for 30 min at 37°C, and the O.D_600_ value was used to measure its residual activity.

The effects of temperature, pH, and NaCl on the antimicrobial activity of LysECP26 were assessed by a turbidity reduction assay [26, 27]. LysECP26 was inoculated into the EDTA-treated *E. coli* DH5α cells (final conc. 1,000 ng/ml) and incubated for 5 min under the following conditions: temperature (4°C, 25°C, 37°C, 42°C, 55°C, and 72°C), pH (0.1 M HCl-KCl buffer for pH 2.0, 50 mM sodium acetate for pH 3.6–5.6 and 20 mM Tris-HCl for pH 7.0–9.0), NaCl (0, 100, 500, 750, 1,000 mM). After incubation, the O.D_600_ value was determined.

### STEC Inactivation by LysECP26 with OMPs

Evaluation of STEC inactivation was carried out using a modified method from previous reports [17, 27]. EDTA, citric acid, and lactic acid were used as OMPs. Exponentially grown *E. coli* 13930 (O157:H7) culture was washed and diluted to a final density of 10^7^ CFU/mL with reaction buffer. *E. coli* (50 μl) was incubated for 30 min at 37°C with 25 μl of LysECP26 (final conc. 40 ng/μl) containing 25 μl OMPs (final conc. 5 mM). Distilled water (DW) was used as a negative control. After incubation, samples were serially diluted and spread on SMAC agar (Oxoid, UK). After overnight incubation at 37°C, individual colonies were counted.

### Statistical Analysis

The experiments were replicated three times, and the experimental results are expressed as means ± standard deviations (SD). Data were evaluated using a one-tailed t-test. The data were analyzed using SPSS ver. 25 (SPSS Inc., USA).

## Results and Discussion

### Bioinformatic Analysis of Endolysin LysECP26

**Fig. 1:**
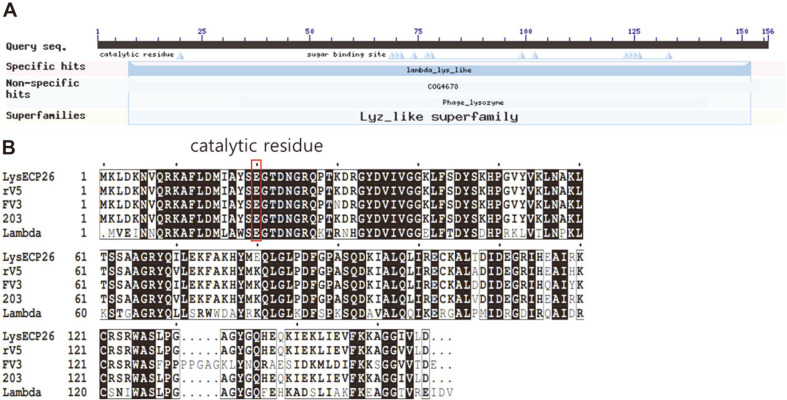
Bioinformatic analysis of endolysin LysECP26. (**A**) NCBI conserved domain database (CDD)-based annotation of the endolysin LysECP26 (**B**) Sequence alignment of various *E. coli* phage endolysin; vB_EcoM-ECP26 endolysin, rV5 phage endolysin, phage FV3 endolysin, phage 203 endolysin, phage Lambda endolysin. Red box means common catalytic residues.

**Fig. 2:**
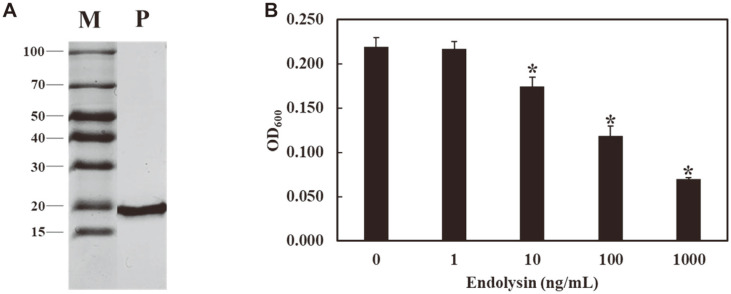
Purification and evaluation of lytic activity of endolysin LysECP26. (**A**) SDS-PAGE of the samples from different purification steps. M; standard molecular wight marker, P; purified remcombinant endolysin. (**B**) Antimicrobial activity of purified LysECP26. Different concentration of LysECP26 (1, 10, 100, 1,000 mg/ml) were added to *E. coli* DH5α cell pellets. The data were presented as mean ± SD (*n* = 3). The asterisk indicate significant differences; *p < 0.01.

With a few exceptions, most phage endolysins from gram-negative bacteria are composed of a globular protein with a single domain [28]. The endolysin of the phage vB_EcoM-ECP26, named LysECP26, is a 156-amino-acid protein with a molecular mass of 17.5 kDa and a lysozyme-like (*N*-acetylmuramidase) catalytic domain (Fig. 1A). Endolysins derived from gram-negative infecting phages are usually unable to pass through OM; however, they are known to be more effective than commercial Hen-egg white lysozyme, allowing for its use as a more efficient pathogen control [29]. According to the BLASTP result, LysECP26 showed higher homology with lambda lysozyme (62.42%) than with T4 lysozyme (33.33%). Additionally, LysECP26 was revealed to be almost identical with the endolysin of rV5 (98.08%) (data not shown). Alignment of rV5-like phage endolysins with phage lambda endolysins showed that the protein size and most residues (black boxes) are similar, but many mutations have also occurred (white boxes) (Fig. 1B). Nonetheless, all five phages have a common catalytic residue of Glu20 (red box). These data suggest that the rV5-like phage endolysin containing LysECP26 probably shares a similar lysis mechanism with lambda phage endolysin, but the stability or activity of the enzyme might be different. To evaluate the lytic activity of LysECP26, transformation and overexpression were attempted using the strain *E. coli* BL21(DE3). However, due to the toxicity of LysECP26, cell growth was inhibited, and the purification could not proceed (data not shown). To solve this problem, the strain was changed to *E. coli* C41(DE3), which is specialized for toxic protein overexpression, and the LysECP26 was successfully purified (approximately 18.33 kDa) (Fig. 2A).

### Antimicrobial Activity and Spectrum of LysECP26

To assess the bactericidal activity of LysECP26, purified LysECP26 was serially diluted and treated with OM-removed *E. coli* DH5α cells (Fig. 2C). LysECP26 revealed lytic activity dose-dependently, but the use of 10 ng/ml drastically dropped the lytic activity, and the use of 1 ng/ml did not show any lytic activity. In addition, treatment with an excess of 1,000 ng/ml of LysECP26 produced the same turbidity-reducing effect, and no lytic activity was observed when EDTA was not included in the treatment (data not shown). Therefore, the minimal bactericidal activity (MBC) of LysECP26 was determined to be 10 ng/ml, and a dose greater than 1,000 ng/ml with OMPs should be used to yield potent bactericidal effects.

To identify the antimicrobial spectrum of LysECP26, a turbidity reduction assay was performed against six gram-positive strains and 29 gram-negative strains (Table 1). The gram-negative strains were all dissolved by LysECP26 with in 30 min. However, all six gram-positive strains were not lysed (Table S1). The lysis spectrum of LysECP26 resembled previous reports of *Pseudomonas aeruginosa* phage endolysin LysPA26, which had one catalytic domain without a signal-anchor-release domain [25]. This selective and strong lytic activity against various gram-negative bacteria, as well as *E. coli* O157:H7, suggests the possible use of LysECP26 as an antimicrobial agent.

### Biochemical Properties of LysECP26

**Fig. 3:**
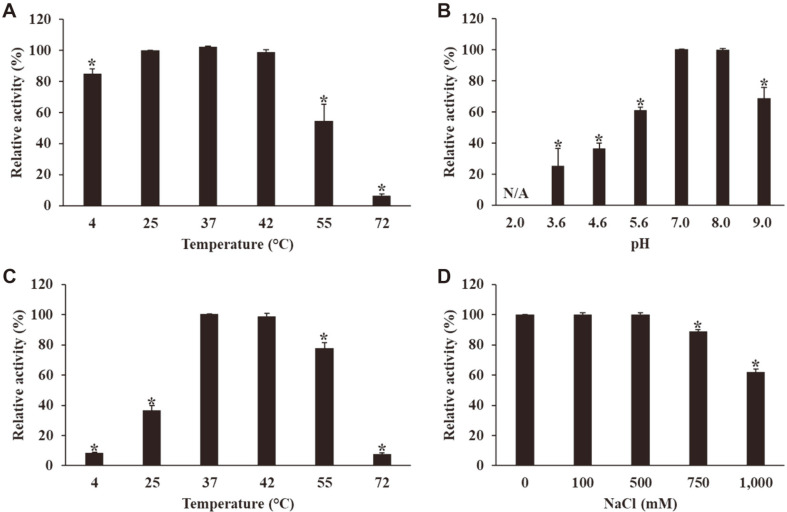
Characterization of LysECP26 at different conditions. (**A**) Thermal stability of LysECP26. The effects at various pH (**B**), temperatures (**C**), and NaCl concentrations (**D**) on the lytic activity of LysECP26. The data were presented as mean ± SD (*n* = 3). The asterisk indicate significant differences; *p < 0.01.

The thermal stability of LysECP26 was assessed under various temperatures from 4°C to 72°C for 1 h (Fig. 3A). LysECP26 remained stable without losing activity from 25°C to 42°C and maintained 80% residual activity at 4°C. However, when heated to 55°C, LysECP26 activity dropped below 55%, and it completely disappeared when heated to 72°C. These results resemble the thermal stability tendency of T4 phage endolysin. T4 phage endolysin also maintained 100% residual activity at around 37°C, but activity decreased as the temperature increased above 50°C [30].

LysECP26 activity was also determined at the various temperatures (4°C–72°C), pH (2.0–9.0), and NaCl (0–1,000 mM) conditions. At 37°C and 42°C, it showed normal activity as well as temperature stability, whereas at 4°C, 25°C, and 72°C, the activity dropped sharply by 8%, 37%, and 8%, respectively (Fig. 2C). Interestingly, it showed 78% activity at 55°C, which was in contrast to the low enzyme stability at 55°C. Based on these results, the thermal stability and optimal temperature of LysECP26 are distinguished, and a temperature of 37°C–42°C is optimal for the enzyme. In pH 7.0–8.0 conditions, LysECP26 showed normal activity without any decrease, but in acidic condition below pH 5.6, activity decreased in proportion to the change in pH, and was completely lost at pH 2.0. Under basic conditions at pH 9.0, it showed 69% activity, which was higher than that under acidic conditions (Fig. 3B). The addition of NaCl did not affect the enzyme efficacy, and the addition of over 750 mM of NaCl decreased the activity (Fig. 3D). LysPA26 and SPN9CC endolysin, two previously studied endolysins, showed the highest activity at neutral pH and around 37°C, and decreased activity at low pH and high temperature [25, 27]. While it was common for NaCl supplementation to increase endolysin activity, LysECP26 experienced no effects, proving to be different from other endolysins in this regard. LysPA26, an endolysin derived from *Pseudomonas* phage, also was not significantly affected by the concentration of NaCl [25].

### STEC Inactivation by LysECP26 with OMPs

**Fig. 4:**
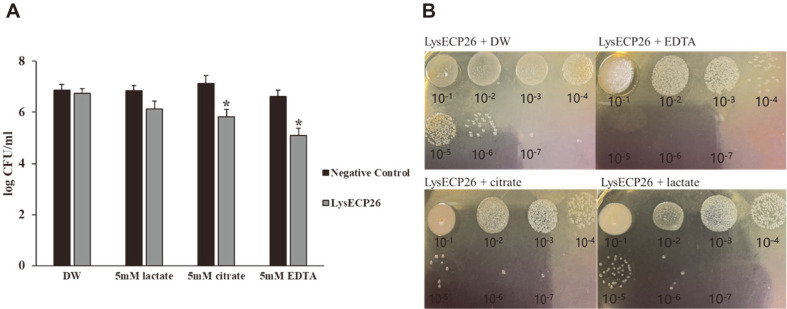
Inactivation of *E. coli* O157:H7 by LysECP26 with outermembrane permeabilizers (OMPs). (**A**) Reduction of *E. coli* O157:H7 after treatment of LysECP26 with DW, lactate, citrate, and EDTA at 37°C for 30 min. The data were presented as mean ± SD (*n* = 3). The asterisk indicate significant differences; *p < 0.01. (**B**) Visualization of the combinational effect of LysECP26 and OMPs against *E. coli* O157:H7. After the reaction, 10 μl of sequential dilutions were spotted on the SMAC plates. The numbers under the colony represent the dilution ratio.

Gram-negative bacteria, including *E. coli* O157:H7, possess an outer membrane that prevents penetration by endolysin [31]. This is one of the barriers to the application of endolysin in targeting gram-negative bacteria, and to overcome this, treatment with three types of OMPs (EDTA, citrate, lactate) was studied in combination with LysECP26 against *E. coli* O157:H7 (Figs. 4A and 4B). When LysECP26 was treated with DW, the *E. coli* population showed no significant decrease compared with the initial concentration (7 log CFU/ml). Moreover, treatment with OMPs or DW alone also did not show a significant bactericidal effect (*p* > 0.01). LysECP26 with 5 mM EDTA produced a killing effect of 1.44 log CFU/ml compared with treatment with 5 mM EDTA alone, and the combination of 5 mM citrate and LysECP26 produced a killing effect of 1.14 log CFU/ml compared with treatment with 5 mM citrate alone. However, the combination of 5 mM lactate and LysECP26 only showed a reduction effect of 0.62 log CFU/ml (*p* > 0.01) compared with treatment with 5 mM lactate alone. EDTA is one of the most well-known OMPs that can act as a chelating agent and has been studied in combination with many endolysins. EDTA acts as a powerful OMP, as seen in these experimental results, but due to its cytotoxicity, it is only possible to use very small amounts as food additives [32]. Therefore, organic acid was selected as a new OMP candidate. In a recent study, significant control effects of pathogens were confirmed by treating lysin ABgp46 from *Acinetobacter* phage and lysin Lys68 from *Salmonella* phage together with organic acids such as citric acid, malic acid, and lactic acid as the OMPs [17, 33]. Generally, the bactericidal effect of organic acid is explained by hydrogen ions according to pKa [34, 35]. The combination of lysin ABgp46 with organic acid was most effective against *E. coli* O157:H7 when the pH was low and the organic acid had a low pKa [33].

The activity of LysECP26 was stronger when mixed with citric acid (pKa 3.13) than that with lactic acid (pKa 3.86). These synergistic effects suggested that the combination of organic acid and LysECP26 could be an excellent *E. coli* O157:H7 control agent. Although the combination of OMPs and endolysin inhibited *E. coli* O157:H7 to a significant level, actual food application will have limitations because it is difficult to implement buffer conditions. Therefore, there is a need to find strong OMPs that do not affect the sensory properties of foods other than EDTA or organic acid. Candidate OMPs to consider are essential oils that target OM such as carvacrol and thymol [36]. A recent study also reported that the viability of *E. coli* was reduced by 3 log units by combined action of endolysin Cpl-7S and carvacrol [37].

In this study, an endolysin of *E. coli* phage belonging to the rV5-like phage genus was identified and purified. This endolysin had a lysozyme-like catalytic domain but was very different from the sequence of lambda phage endolysin. Furthermore, LysECP26 exhibited strong activity with a broad lytic spectrum against various gram-negative strains and had moderate activity in various environmental conditions. Although LysECP26 was limited in that it could not pass through OM, *E. coli* O157: H7 could be effectively controlled by combining LysECP26 with OMPs such as EDTA and organic acids. Therefore, LysECP26 could be potentially used as an effective biocontrol agent for gram-negative strains, including *E. coli* O157: H7.

## Supplemental Materials



Supplementary data for this paper are available on-line only at http://jmb.or.kr.

## Figures and Tables

**Table 1:** Lysis spectrum of endolysin LysECP26 for various bacteria including *E. coli*.

Bacteria	Positive	Total
*Bacillus* spp.	0	3
*Lactobacillus plantarum*	0	1
*Staphylococcus aureus*	0	1
*Enterococcus faecalis*	0	1
*Salmonella* spp.	4	4
*Cronobacter sakazakii*	1	1
*Klebsiella pneumoniae*	1	1
*Escherichia coli* O157:H7	5	5
*Escherichia coli* Non-O157	18	18

## References

[1] Conway T, Krogfelt KA, Cohen PS (2004). The life of commensal *Escherichia coli* in the mammalian intestine. EcoSal Plus.

[2] Hunt JM (2010). Shiga toxin-producing *Escherichia coli* (STEC). Clin. Lab. Med..

[3] Yang SC, Lin CH, Aljuffali IA, Fang JY (2017). Current pathogenic *Escherichia coli* foodborne outbreak cases and therapy development. Arch. Microbiol..

[4] Pirnay JP, Verbeken G, Ceyssens PJ, Huys I, De Vos D, Ameloot C (2018). The magistral phage. Viruses.

[5] Lin DM, Koskella B, Lin HC (2017). Phage therapy: an alternative to antibiotics in the age of multi-drug resistance. World J. Gastrointest. Pharmacol. Ther..

[6] Viazis S, Akhtar M, Feirtag J, Brabban AD, Diez-Gonzalez F (2011). Isolation and characterization of lytic bacteriophages against enterohaemorrhagic *Escherichia coli*. J. Appl. Microbiol..

[7] Ferguson S, Roberts C, Handy E, Sharma M (2013). Lytic bacteriophages reduce *Escherichia coli* O157: H7 on fresh cut lettuce introduced through cross-contamination. Bacteriophage.

[8] McLean SK, Dunn LA, Palombo EA (2013). Phage inhibition of *Escherichia coli* in ultrahigh-temperature-treated and raw milk. Foodborne Pathog. Dis..

[9] Magnone JP, Marek PJ, Sulakvelidze A, Senecal AG (2013). Additive approach for inactivation of *Escherichia coli* O157:H7, *Salmonella*, and *Shigella* spp. on contaminated fresh fruits and vegetables using bacteriophage cocktail and produce wash. J. Food Prot..

[10] Kropinski AM, Waddell T, Meng J, Franklin K, Ackermann H-W, Ahmed R (2013). The host-range, genomics and proteomics of *Escherichia coli* O157:H7 bacteriophage rV5. Virol. J..

[11] Truncaite L, Šimoliūnas E, Zajančkauskaite A, Kaliniene L, Mankevičiūte R, Staniulis J (2012). Bacteriophage vB_EcoM_FV3: a new member of "rV5-like viruses". Arch. Virol..

[12] Kim M, Heu S, Ryu S (2014). Complete genome sequence of enterobacteria phage 4MG, a new member of the subgroup "PVP-SE1-like phage" of the "rV5-like viruses". Arch. Virol..

[13] Korf IHE, Meier-Kolthoff JP, Adriaenssens EM, Kropinski AM, Nimtz M, Rohde M (2019). Still something to discover: novel insights into *Escherichia coli* phage diversity and taxonomy. Viruses.

[14] Schmelcher M, Loessner MJ (2016). Bacteriophage endolysins: applications for food safety. Curr. Opin. Biotechnol..

[15] Shabbir MAB, Hao H, Shabbir MZ, Wu Q, Sattar A, Yuan Z (2016). Bacteria vs. bacteriophages: parallel evolution of immune arsenals. Front. Microbiol.

[16] Nelson DC, Schmelcher M, Rodriguez-Rubio L, Klumpp J, Pritchard DG, Dong S (2012). Endolysins as antimicrobials. Adv. Virus Res..

[17] Oliveira H, Thiagarajan V, Walmagh M, Sillankorva S, Lavigne R, Neves-Petersen MT (2014). A thermostable *Salmonella* phage endolysin, Lys68, with broad bactericidal properties against gram-negative pathogens in presence of weak acids. PLoS One.

[18] Park DW, Lim GY, Lee YD, Park JH (2020). Characteristics of lytic phage vB_EcoM-ECP26 and reduction of shiga-toxin producing *Escherichia coli* on produce romaine. Appl. Biol. Chem..

[19] Altschul SF, Gish W, Miller W, Myers EW, Lipman DJ (1990). Basic local alignment search tool. J. Mol. Biol..

[20] Lu S, Wang J, Chitsaz F, Derbyshire MK, Geer RC, Gonzales NR (2020). CDD/SPARCLE: the conserved domain database in 2020. Nucleic Acids Res..

[21] Sievers F, Wilm A, Dineen D, Gibson TJ, Karplus K, Li W (2011). Fast, scalable generation of high-quality protein multiple sequence alignments using clustal omega. Mol. Syst. Biol..

[22] Dumon-Seignovert L, Cariot G, Vuillard L (2004). The toxicity of recombinant proteins in *Escherichia coli*: a comparison of overexpression in BL21(DE3), C41(DE3), and C43(DE3). Protein Exp. Purif..

[23] Wu M, Hu K, Xie Y, Liu Y, Mu D, Guo H (2018). A novel phage PD-6A3, and its endolysin Ply6A3, with extended lytic activity against *Acinetobacter baumannii*. Front. Microbiol..

[24] Lim JA, Shin H, Kang DH, Ryu S (2012). Characterization of endolysin from a *Salmonella* Typhimurium-infecting bacteriophage SPN1S. Res. Microbiol..

[25] Guo M, Feng C, Ren J, Zhuang X, Zhang Y, Zhu Y (2017). A novel antimicrobial endolysin, LysPA26, against *Pseudomonas aeruginosa*. Front. Microbiol..

[26] Yu JH, Lim JA, Chang HJ, Park JH (2019). Characteristics and lytic activity of phage-derived peptidoglycan hydrolase, LysSAP8, as a potent alternative biocontrol agent for *Staphylococcus aureus*. J. Microbiol. Biotechnol..

[27] Lim JA, Shin H, Heu S, Ryu S (2014). Exogenous lytic activity of SPN9CC endolysin against gram-negative bacteria. J. Microbiol. Biotechnol..

[28] Schmelcher M, Donovan DM, Loessner MJ (2012). Bacteriophage endolysins as novel antimicrobials. Future Microbiol..

[29] Briers Y, Lavigne R, Volckaert G, Hertveldt K (2007). A standardized approach for accurate quantification of murein hydrolase activity in high-throughput assays. J. Biochem. Biophys. Methods.

[30] Tsugita A, Inouye M (1968). Purification of bacteriophage T4 lysozyme. J. Biol. Chem..

[31] Briers Y, Lavigne R (2015). Breaking barriers: expansion of the use of endolysins as novel antibacterials against Gram-negative bacteria. Future Microbiol..

[32] Heimbach J, Rieth S, Mohamedshah F, Slesinski R, Samuel-Fernando P, Sheehan T (2000). Safety assessment of iron EDTA [sodium iron (Fe3+) ethylenediaminetetraacetic acid]: summary of toxicological, fortification and exposure data. Food Chem. Toxicol..

[33] Oliveira H, Vilas Boas D, Mesnage S, Kluskens LD, Lavigne R, Sillankorva S (2016). Structural and enzymatic characterization of ABgp46, a novel phage endolysin with broad anti-gram-negative bacterial activity. Front. Microbiol..

[34] Alakomi HL, Skyttä E, Saarela M, Mattila-Sandholm T, Latva-Kala K, Helander IM (2000). Lactic acid permeabilizes gram-negative bacteria by disrupting the outer membrane. Appl. Environ. Microbiol..

[35] Lu HJ, Breidt F, Pérez-Díaz IM, Osborne JA (2011). Antimicrobial effects of weak acids on the survival of *Escherichia coli* O157:H7 under anaerobic conditions. J. Food Prot..

[36] Hyldgaard M, Mygind T, Meyer RL (2012). Essential oils in food preservation: mode of action, synergies, and interactions with food matrix components. Front. Microbiol..

[37] Díez-Martínez R, de Paz H, Bustamante N, García E, Menéndez M, García P (2013). Improving the lethal effect of Cpl-7, a *Pneumococcal* phage lysozyme with broad bactericidal activity, by inverting the net charge of its cell wall-binding module. Antimicrob. Agents Chemother..

