# Supplementary material for: Characterization of Endolysin LysECP26 Derived from rV5-like Phage vB_EcoM-ECP26 for Inactivation of Escherichia coli O157:H7
Source: J Microbiol Biotechnol. 2020 Jul 17;30(10):1552–8. doi: 10.4014/jmb.2005.05030 (PMC9728275; doi:10.4014/jmb.2005.05030)
Supplement: Supplementary file 1 [file JMB-30-10-1552-supple.pdf]

J. Microbiol. Biotechnol.
https://doi.org/10.4014/jmb.2005.05030

Table S1. Bacterial strains used in this study.

	Bacterial strain	Source or reference	lysis activity[a]
Gram positive strain	*Bacillus cereus* 40935	KCCM	-
	Bacillus subtilis 14593	ATCC	-
	Bacillus thuringensis 1510	KCTC	-
	Lactobacillus plantarum 8014	ATCC	-
	Staphylococcus aureus RN4220	LAB	-
	Enterococcus faecalis 3206	KCTC	-
Gram negative strain	*Salmonella typhimurium* 14028	ATCC	+
	Salmonella typhimurium 1A	WT	+
	Salmonella enterotidis Egg isolate	WT	+
	Salmonella enterotidis Chicken isolate	WT	+
	Cronobacter sakazakii 2949	KCTC	+
	klebsiella pneumoniae 13883	ATCC	+
	Escherichia coli 13899 (O157:H7)	NCCP	+
	Escherichia coli 13930 (O157:H7)	NCCP	+
	Escherichia coli 13919 (O157:H7)	NCCP	+
	Escherichia coli 13921 (O157:H7)	NCCP	+
	Escherichia coli 13899 (O157:H7)	NCCP	+
	Escherichia coli 13916 (O117)	NCCP	+
	Escherichia coli 13937 (O103)	NCCP	+
	Escherichia coli 14018 (O91)	NCCP	+
	Escherichia coli 13970 (O111)	NCCP	+
	Escherichia coli 13927 (O55)	NCCP	+
	Escherichia coli 13934 (O179)	NCCP	+
	Escherichia coli 13987 (O55)	NCCP	+
	Escherichia coli 13979 (O104)	NCCP	+
	Escherichia coli 13988 (O104)	NCCP	+
	Escherichia coli 13999 (O104)	NCCP	+
	Escherichia coli 15961 (O26)	NCCP	+
	Escherichia coli 15960 (O55)	NCCP	+
	Escherichia coli NCCP 15959 (O5)	NCCP	+

	***Escherichia coli* NCCP 15962 (O21)**	**NCCP**	+
	***Escherichia coli* NCCP 15958 (O22)**	**NCCP**	+
	***Escherichia coli* DH5α**	**LAB**	+
	***Escherichia coli* BL21(DE3)**	**Invitrogen**	+
	***Escherichia coli* C41(DE3)**	**Novagen**	+

*ATCC, American Type Culture Collection; NCCP, National Culture Collection for Pathogens; KCCM, Korean Culture Center of Microorganisms; KCTC, Korean Collection for Type Cultures, WT; Wild Type, LAB; Laboratory stroage

[a] lysis activity when significant statistical results appeared. +; $p<0.01$. -; $p>0.01$.
